# Lactate-Driven Biphasic Reprogramming of Macrophage Immune Responses

**DOI:** 10.3390/metabo16100788

**Published:** 2026-10-16

**Authors:** Qingshuang Guo, Pan Ren, Lanqi He, Jiaying Gao, Ruiqi Lu, Yitian Wang, Zhiheng Zhang, Xiaohui Han, Xi Zhang, Chao Fang

**Affiliations:** 1 School of Medicine, Northwest University, Xi’an 710069, China; 15638097898@163.com (Q.G.); 17764810366@163.com (R.L.); 13021865128@163.com (X.H.); 2 Department of Plastic and Reconstructive Surgery, Xijing Hospital, Fourth Military Medical University, Xi’an 710032, China; hlq970704@163.com (L.H.); 17398622366@163.com (J.G.); 3 Department of Burns, Plastic and Wound Repair Surgery, the Second Affiliated Hospital, Xi’an Jiaotong University, Xi’an 710004, China; renpan1008@xjtu.edu.cn; 4 Department of Pharmacology, School of Pharmacy, Fourth Military Medical University, Xi’an 710032, China; 13571825655@163.com (Y.W.); zhangzhiheng1106@163.com (Z.Z.)

**Keywords:** lactate, lactylation, immunometabolism, macrophage plasticity, immune regulation

## Abstract

Lactate is a metabolic substrate, an extracellular signaling molecule, and a precursor for protein lactylation, with diverse effects on macrophage immunity. Evidence from infection, tissue injury, efferocytosis, and cancer indicates that lactate-associated pathways can amplify inflammation, support resolution and tissue repair, or sustain immune suppression. These outcomes depend on the timing, cellular source and compartment, chemical formulation and pH, transport and receptor engagement, and the chemistry and targets of lactylation. In this narrative review, we critically synthesize the biochemical foundations of lactate metabolism and lactyl-group donation and examine the mechanisms underlying these distinct macrophage responses. We distinguish model-specific biphasic responses from a universal sequence of early inflammation followed by resolution, and separate reparative programs from impaired antimicrobial or antitumor immunity. We also evaluate evidence for histone and non-histone lactylation, emphasizing the limits of metabolic perturbations and chromatin associations for establishing site-specific causality. This framework identifies experimental variables that should be controlled and supports context-selective investigation of lactate-related therapeutic targets in inflammatory diseases and cancer.

## 1. Introduction

Macrophages participate in pathogen clearance, inflammatory mediator release, apoptotic-cell clearance, and tissue repair. This broad functional spectrum requires high phenotypic plasticity, enabling macrophages to mount robust inflammatory responses and subsequently transition to inflammation resolution and tissue restoration [1,2,3]. This plasticity is intimately coupled to immunometabolic reprogramming, wherein dynamic shifts between glycolysis and mitochondrial oxidative metabolism alter intracellular metabolite levels to dictate macrophage fate [4,5,6]. Furthermore, among the various mechanisms by which metabolism influences cell function, metabolite-driven protein post-translational modifications have emerged as a crucial regulatory layer [7,8].

Lactate, the conventional end product of glycolysis, has been redefined from a passive metabolic waste into an active immunoregulatory molecule [9,10]. Accumulating evidence has revealed its diverse and complex roles in shaping the fates and functions of immune cells, particularly in macrophages and dendritic cells [11,12,13]. A paradigm shift occurred with the discovery that lactate serves as a precursor for histone lactylation, directly linking metabolic flux to chromatin remodeling and the expression of repair-associated genes in macrophages [14]. However, the immunological effects of lactate remain debated. Across experimental models, lactate-associated pathways can amplify early inflammatory injury, support pro-resolving and reparative macrophage programs, or sustain tumor-associated immune suppression [15,16,17,18,19]. We propose that these apparently divergent observations are not mutually exclusive but instead reflect the context dependence of lactate biology. The resulting immune response is shaped by the inflammatory phase, cellular source and intracellular versus extracellular compartment, receptor and transporter engagement, and the chemistry and stereochemistry of lactyl-group donors and resulting lysine modifications [17,20,21,22,23]. These findings support context-dependent regulation by lactate, with biphasic responses demonstrated in particular experimental settings.

In this narrative review, we revisit the biochemical foundations of lactate metabolism and lactylation and critically synthesize the mechanisms underlying inflammatory amplification, resolution, tissue repair, and tumor-associated immune suppression. We consider temporal regulation alongside lactate source, compartment, chemical form, pH, and downstream effector mechanisms. Finally, we address controversies and methodological challenges to define the evidence and experimental controls needed for investigating lactate-related therapeutic targets.

## 2. Biological Basis of Lactate and Lactylation

### 2.1. Sources, Intracellular Fate, and Microenvironmental Concentrations of Lactate

Lactate is generated from glycolytic pyruvate via lactate dehydrogenase (LDH). In macrophages, intracellular lactate accumulation primarily results from enhanced glycolytic flux, where LDHA catalyzes pyruvate reduction. Alternatively, macrophages can acquire lactate from the extracellular milieu, a process heavily dependent on proton-coupled monocarboxylate transporters (MCTs) [24,25]. This dynamic exchange of lactate, often termed the “lactate shuttle”, is dictated by the interplay of concentration gradients, cellular metabolic demand, and tissue context [24]. MCT1 commonly facilitates lactate uptake and reutilization, whereas MCT4 supports efflux from highly glycolytic cells. These are context-dependent net fluxes: proton and substrate gradients govern transport, and MCT1 can also mediate lactate export. MCT2 (SLC16A7) is a high-affinity lactate transporter [26]. Its functional involvement has been reported in murine bone marrow macrophages undergoing osteoclast differentiation induced by receptor activator of nuclear factor κB ligand (RANKL) [27], but these data do not establish its role in inflammatory or reparative macrophage lactate transport. MCT3 (SLC16A8) is preferentially expressed in retinal pigment and choroid plexus epithelia [28]; its contribution to lactate handling in the macrophage states discussed here remains to be established. MCT3 transcripts were not detected in the macrophage-osteoclast system tested by Imai et al. [27].

Regardless of whether it is endogenously synthesized via glycolysis or imported from the extracellular milieu, intracellular lactate acts as an active metabolic and signaling hub rather than a passive waste product. Among its diverse biological effects, lactate influences macrophage function through several interconnected pathways. In terms of metabolic homeostasis, its continuous interconversion with pyruvate via LDH is coupled to the intracellular NAD^+^/NADH redox balance [24,25], while lactate-derived pyruvate can fuel the mitochondrial tricarboxylic acid cycle in defined macrophage states [29]. Furthermore, in the context of early infection or metabolic stress, previous studies have reported that elevated intracellular lactate can promote reactive oxygen species (ROS) production, thereby potentiating inflammatory outputs [17]. Beyond ROS generation, lactate influences mitochondrial function in additional ways. During alternative activation, carbon derived from extracellular lactate enters the mitochondrial tricarboxylic acid cycle after conversion to pyruvate, thereby supporting oxidative metabolism and polarization; blocking mitochondrial pyruvate uptake blunts these effects [29]. In sepsis models, deficient Spinster homolog 2 (SPNS2)/Sphingosine-1-Phosphate (S1P) signaling increased prostaglandin E2 (PGE2)-EP4 activation, impaired the malate-aspartate shuttle and mitochondrial respiration, and thereby promoted intracellular lactate accumulation and ROS-associated inflammatory injury [17,30] (Section 4.1). Macrophage studies have reported a mitochondrial pyruvate carrier (MPC)-sensitive lactate-derived pyruvate route [29] and, more recently, MCT1 localization together with perturbation-associated changes in mitochondrial lactate signals and respiration [31]. However, the latter evidence does not resolve the compartment-specific flux of lactate oxidation. Additionally, lactate can supply enzymatic lactyl-group donor pathways, while the methylglyoxal–glyoxalase pathway provides a chemically distinct lactoyl donor route [32,33,34,35].

Accurately evaluating the biological functions of lactate requires careful consideration of its concentration across different microenvironments. While plasma lactate is maintained at approximately 1 to 2 mM under physiological conditions, it frequently exceeds 4 mM in sepsis and can reach 10 to 40 mM locally in ischemic tissues and tumors [9,10]. Although most in vitro studies employ 10 to 25 mM lactate to mimic these pathological states, it is crucial to recognize that in vitro medium acidification differs fundamentally from in vivo buffering systems. Therefore, extrapolating these experimental findings requires strict caution to ensure that the applied concentration and pH combinations genuinely reflect the target pathological context.

### 2.2. Writers, Erasers, and Potential Donors of Lactylation

Histone lactylation, a form of lysine lactylation (Kla), is a post-translational modification in which a lactyl group is covalently attached to lysine residues on histones [14,36]. Using high-resolution mass spectrometry (MS), Zhang et al. [14] identified multiple histone lactylation sites in macrophages and identified histone H3 lysine 18 lactylation (H3K18la) as a representative mark. At the molecular level, this process is dynamically and reversibly regulated by specific enzymes. Lysine acetyltransferases (KATs), including p300 and CREB-binding protein (CBP), can catalyze lactyl-group transfer in defined biochemical and cellular settings and are commonly described as “writers”. Conversely, histone deacetylases 1-3 (HDAC1-3) and sirtuin 2 (SIRT2) exhibit delactylase activity, acting as “erasers” that remove these modifications [37,38].

Lactyl-CoA is a demonstrated donor for acyltransferase-catalyzed lactylation, but the contribution of this route relative to alternative mechanisms remains context-dependent [14,22,39]. Recent studies in specific tumor models indicate that nuclear metabolic enzymes can supply these donors locally. For instance, guanosine triphosphate-specific succinyl-CoA synthetase (GTPSCS) and acyl-coenzyme A synthetase short-chain family member 2 (ACSS2) can facilitate the generation of lactyl-CoA within the nucleus. Furthermore, ACSS2 can cooperate directly with lysine acetyltransferase 2A (KAT2A) to drive lactyl transfer, supporting a role for local donor production in these models [32,33]. The relative contributions of these routes in macrophages remain to be established.

Alongside lactyl-CoA-dependent enzymatic lactylation, a non-enzymatic modification pathway mediated by lactoylglutathione has recently emerged [23,34,35]. In this alternative route, methylglyoxal (MGO), a glycolytic by-product, spontaneously forms a hemithioacetal intermediate with glutathione (GSH). This intermediate is subsequently converted by glyoxalase 1 (GLO1) into S-D-lactoylglutathione (LGSH, also denoted SLG), which is then hydrolyzed by glyoxalase 2 (GLO2) to generate D-lactate and GSH. However, when GLO2 activity decreases or LGSH accumulates, LGSH can serve as a highly reactive donor, promoting non-enzymatic lysine lactoylation through an S-to-N acyl transfer [34].

This pathway also highlights the biological significance of lactate stereoisomers. Mammalian cells predominantly produce L-lactate, whereas cellular D-lactate can be generated through methylglyoxal detoxification [23,34]. Throughout this review, lysine L-lactylation and D-lactylation (also termed D-lactoylation) are distinguished explicitly when stereochemistry has been resolved. The terms lactylation and lactoylation alone do not establish stereochemistry or donor origin; for unresolved modifications, the reported terminology is retained without assigning a stereoisomer. Unless otherwise specified, lactate refers to L-lactate.

## 3. Macrophage Metabolic Reprogramming and Lactylation

### 3.1. Metabolic Reprogramming of Macrophages

Macrophages adapt glycolytic and mitochondrial metabolism to their activation state, tissue environment, and functional demands [29,40]. In commonly used murine inflammatory models, increased glycolysis and redistribution of glucose flux through the pentose phosphate pathway are early responses [41]. With sustained stimulation, tricarboxylic acid cycle remodeling includes itaconate synthesis from cis-aconitate [42] and altered succinate metabolism [43,44]. Later induction of nitric oxide production can further suppress mitochondrial respiration in these models. These processes overlap, and their timing depends on the stimulus and macrophage population. In many models of pathogen exposure or inflammatory stimulation, increased aerobic glycolysis supports ATP production and biosynthesis and is accompanied by intracellular lactate accumulation [45,46,47,48,49]. Remodeling the tricarboxylic acid (TCA) cycle also alters metabolite availability. Succinate accumulation and oxidation can support hypoxia-inducible factor 1α (HIF-1α) signaling and mitochondrial ROS production, thereby promoting inflammatory outputs such as interleukin-1β (IL-1β) [43,50,51]. Oxidative metabolism and fatty acid utilization can support alternative activation and tissue repair in defined settings [52,53,54]. However, this does not establish fatty acid oxidation (FAO) as the predominant fuel pathway in all reparative macrophages. Inflammatory mitochondrial dysfunction can limit subsequent repolarization [55], while efferocytosis can require a transient glycolytic increase to sustain apoptotic-cell clearance [56]. Recent work further distinguished early HIF-1α activity driven by Rubicon (RUBCN) and NADPH oxidase 2 (NOX2) from subsequent inducible nitric oxide synthase (iNOS)/nitric oxide (NO)-mediated suppression of mitochondrial respiration [44]. Thus, glycolytic and oxidative programs contribute to macrophage function through model-specific changes in flux and redox signaling rather than a universally reversible metabolic switch.

### 3.2. Effects of Histone Lactylation on Macrophages

Histone lactylation provides a potential link between glycolytic metabolism and transcriptional regulation [14,36]. Its association with macrophage plasticity has been examined in infection, tissue repair, cancer, and other disease settings [11,57,58,59]. In the original lactate-clock model, histone lactylation increased later than early inflammatory transcription and was associated with the induction of homeostasis-related genes [14,60]. Zhang et al. [14] reported delayed increases in histone lactylation and expression of genes including *Arg1*, supporting a temporal relationship in the macrophage models studied. Subsequent studies linked lactate metabolism and histone lactylation to reparative responses [60]. However, timing and chromatin enrichment alone do not demonstrate that a particular histone lactylation site is necessary or sufficient for gene regulation. The mechanistic evidence and competing explanations are examined in Section 5.2 and Section 6.3 [32,33,36,60].

### 3.3. Non-Histone Lactylation

Beyond chromatin remodeling, non-histone lactylation can regulate protein function at the post-translational level. Unlike histone modifications that primarily govern gene transcription, non-histone lactylation acts directly on target substrates [34,61]. These biochemical changes profoundly impact the modified substrates. Currently, identified non-histone substrates include metabolic enzymes, inflammatory signaling molecules, and stress-related proteins [62]. For instance, the lactylation of metabolic enzymes can directly modulate catalytic activity and metabolic flux, whereas the modification of signaling proteins can alter protein stability, complex assembly, and nucleocytoplasmic translocation [63,64,65,66]. Consequently, non-histone lactylation provides a critical, context-dependent regulatory layer that can alter cellular functions through substrate-specific effects in addition to transcriptional regulation.

## 4. Pro-Inflammatory Effects of Lactate

**Figure 1:**
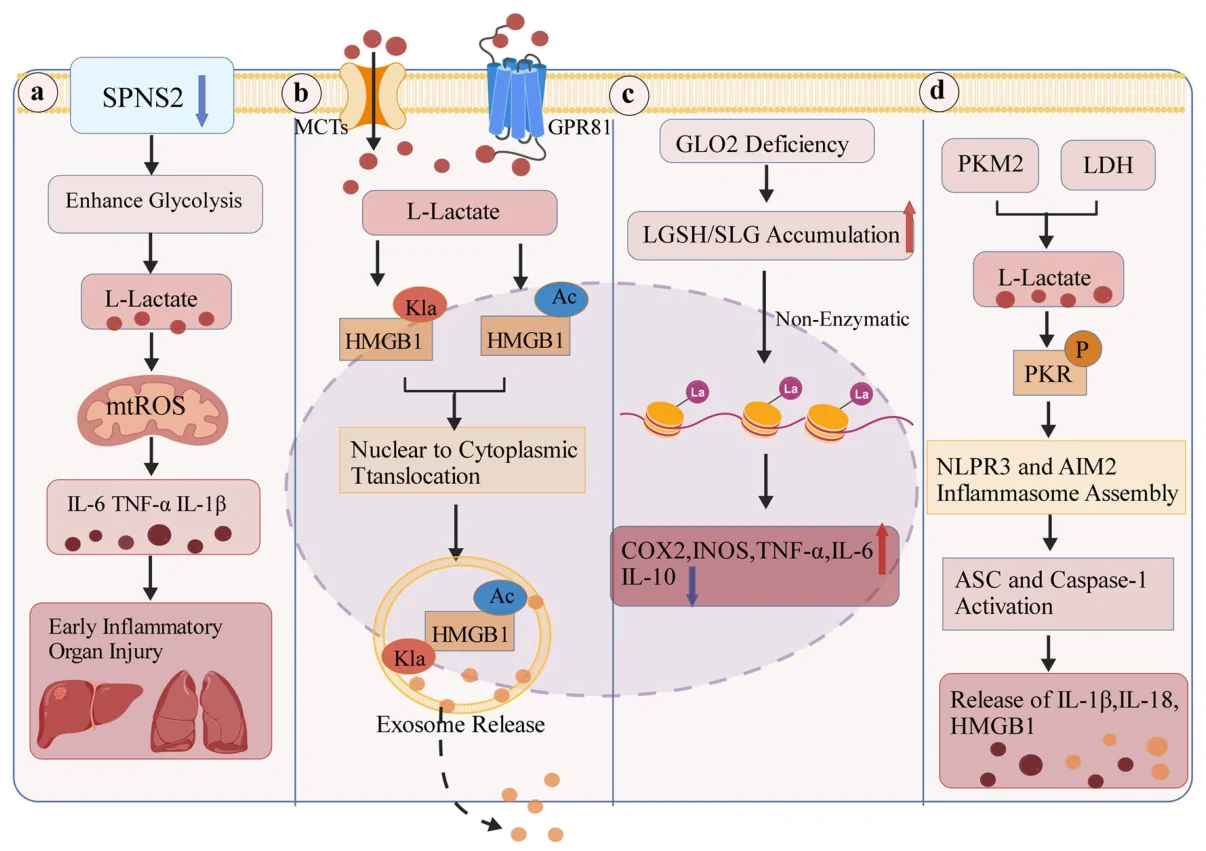
Lactate-related inflammatory pathways in defined macrophage models. (**a**) SPNS2/S1P disruption links lactate accumulation to mitochondrial ROS and sepsis-associated injury. (**b**) Lactate-associated HMGB1 modifications accompany exosomal signaling. (**c**) GLO2 loss alters lactoyl-donor metabolism, histone signals and inflammatory responsiveness. (**d**) PKM2/LDH-dependent fermentation supports PKR and inflammasome responses. Red upward arrows and blue downward arrows denote upregulation and downregulation, respectively. Created with BioGDP (https://biogdp.com/), accessed on 9 October 2026.

Lactate has long been regarded as a passive metabolic waste, closely associated with macrophage polarization toward an anti-inflammatory phenotype and the establishment of an immunosuppressive microenvironment [11,67,68,69,70]. Challenging this classical view, recent studies across various pathological settings indicate that lactate can act as a potent inflammation-amplifying signal. In the following sections, we delineate the specific conditions and underlying mechanisms by which lactate drives pro-inflammatory responses (Figure 1).

### 4.1. The Intracellular Lactate-ROS Axis Drives the Early Hyperinflammatory Response in Sepsis

Fang et al. [17] showed in sepsis models that macrophage SPNS2/S1P signaling supports mitochondrial oxidative phosphorylation and restrains excessive glycolysis, intracellular lactate accumulation, and ROS-associated inflammation. Their follow-up study identified PGE2 as an intermediary: SPNS2/S1P signaling limited PGE2 production through the p38 MAPK pathway, whereas excess PGE2 activated EP4 and impaired the malate-aspartate shuttle and mitochondrial respiration [30]. The resulting lactate accumulation and ROS generation contributed to early inflammatory mediator production and organ injury in these models (Figure 1a) [17,30]. Prolonged SPNS2/S1P disruption also reduced cytokine-producing capacity and antibacterial activity, linking this metabolic disturbance to later immune dysfunction [17,30]. These findings support a model-specific biphasic response; impaired late antimicrobial defense should not be equated with inflammation resolution or tissue repair. Clinical studies have also examined associations of pan-lysine lactylation (pan-Kla), H3K18la, histone H4 lysine 5 lactylation (H4K5la), and histone H3 lysine 56 lactylation (H3K56la) with sepsis stratification or prognosis, but their value as actionable therapeutic targets requires further validation [71,72].

### 4.2. Lactate Promotes HMGB1 Lactylation and Aggravates Organ Injury

Non-histone lactylation has been implicated in lactate-associated inflammatory injury. In a cecal ligation and puncture (CLP) model, Yang et al. [73] found that macrophage uptake of extracellular lactate through MCTs promoted HMGB1 lactylation in a p300/CBP-dependent manner. Lactate exposure was associated with HMGB1 nuclear-to-cytoplasmic translocation and exosomal release. Exosomes from lactate-treated macrophages reduced endothelial VE-cadherin and claudin-5 expression, increased ICAM-1 expression, and impaired barrier integrity [73]. Pharmacological experiments implicated GPR81 signaling through Hippo/YAP-SIRT1 and β-arrestin 2-p300/CBP in lactate-induced HMGB1 acetylation and exosomal release (Figure 1b) [73]. The co-occurrence of lactylation and acetylation warrants distinguishing their respective contributions to HMGB1 trafficking and inflammatory activity. Using site-specific acetylation antibodies, Yang et al. [73] detected increased HMGB1 acetylation signals at K12 and K29, whereas the lactylated residues and their relative occupancy were not determined. Acetylation and lactylation cannot simultaneously occupy the same lysine ε-amino group, but this chemical exclusivity does not demonstrate biological competition. Co-occurrence may involve different lysines or HMGB1 populations; functional cooperation and condition-dependent site preference remain unresolved in this macrophage model [73]. In hypoxic NIH/3T3 fibroblasts, Li et al. [74] reported HMGB1 K177 lactylation by LC-MS/MS and used K177R substitution and knockdown–rescue experiments to link K177 to nuclear export of HMGB1 and T-cell intracellular antigen-related protein (TIAR) and to stress-granule formation. These results do not identify the lactylation site responsible for HMGB1 release in septic macrophages. In a separate study of sepsis-associated acute kidney injury, Wei et al. [75] linked macrophage-derived exosomal HMGB1 to neutrophil mitochondrial DNA release, cGAS/STING signaling, and neutrophil extracellular trap (NET) formation. Together, these studies support lactate-associated HMGB1 modification and exosomal signaling as a route connecting macrophage metabolic changes with endothelial and neutrophil responses.

### 4.3. LGSH-Related Lactoyl Donors Can Enhance Inflammatory Signaling

The LGSH pathway provides an alternative route for protein lactoylation with context-dependent inflammatory consequences. In GLO2-knockout RAW264.7 macrophages, LGSH accumulation was accompanied by increased lactoyl-CoA and altered lactoylation at selected histone sites, without resolving the stereochemistry of the histone modifications. Biochemical experiments showed that LGSH and lactoyl-CoA can donate lactoyl groups for non-enzymatic protein modification [34,35]. GLO2 loss also enhanced responses to the TLR4 agonist Kdo2-lipid A and was associated with altered chromatin accessibility [35]. These findings link donor metabolism, histone modifications, and inflammatory responsiveness, but do not isolate a specific histone D-lactoylation event as the causal mediator (Figure 1c).

### 4.4. Context-Dependent Promotion of NLRP3 Inflammasome Activation by Lactate and Lactic Acid

Lactate metabolism can support NLRP3 inflammasome activation, but the underlying mechanisms depend on whether lactate is produced endogenously or administered exogenously and whether the experimental intervention induces extracellular or intracellular acidification. Current evidence supports two related processes: endogenous lactic acid fermentation facilitates inflammasome activation, whereas intracellular lactic acidification amplifies inflammasome assembly through mitochondrial ROS- and protein kinase R (PKR)-dependent mechanisms.

Evidence from metabolic-intervention studies indicates that endogenous lactic acid fermentation supports NLRP3 inflammasome activation. Lin et al. [76] found that inhibition of glycolysis with 2-deoxy-D-glucose (2-DG), or suppression of lactate production using an LDH inhibitor or *LDHA*-specific siRNA, reduced mature IL-1β and active caspase-1 in macrophages without altering the abundance of NLRP3, ASC, pro-caspase-1, or pro-IL-1β. In nigericin-stimulated THP-1-derived macrophages, the LDH inhibitor also reduced PKR phosphorylation but did not prevent mitochondrial or intracellular ROS production or potassium efflux. These findings suggest that lactic acid fermentation primarily supports inflammasome activation and cytokine processing without detectably increasing the abundance of priming-associated inflammasome components.

Consistent with this metabolic model, Xie et al. [19] linked PKM2-dependent glycolysis to NLRP3 and AIM2 inflammasome activation through PKR. Pharmacological or genetic inhibition of PKM2 or PKR attenuated inflammasome activation and reduced the release of IL-1β, IL-18, and HMGB1 (Figure 1d). The study also examined the effects of exogenously added lactic acid in lipopolysaccharide (LPS)-primed bone marrow-derived macrophages. Because the lactic acid solution was not pH-adjusted, treatment lowered the extracellular pH from 7.4 to 6.6. Under these conditions, lactic acid induced PKR phosphorylation, IL-1β, IL-18, and HMGB1 release, and caspase-1/11 activation, with both NLRP3 and AIM2 contributing to these responses. Thus, these experiments demonstrated a pro-inflammatory effect of exogenously added lactic acid under acidifying conditions but did not isolate the effect of the lactate anion from that of reduced extracellular pH.

The roles of chemical formulation and pH were subsequently examined more directly by Lin et al. [77]. Neither extracellular lactic acid nor HCl alone activated the NLRP3 inflammasome, but both potentiated nigericin- or ATP-induced inflammasome responses. Sodium lactate did not exert a comparable effect at pH 7.4 but became potentiating when the extracellular pH was lowered to 6.9 [77]. These findings indicate that the effects of lactic acid and sodium lactate cannot be interpreted independently of the final extracellular pH. Mechanistically, the accumulation of intracellular lactic acid promoted cytoplasmic acidification and amplified NLRP3 inflammasome assembly through two parallel pathways. One involved mitochondrial dysfunction and ROS production, whereas the other involved PKR phosphorylation, increased PKR-NLRP3 interaction, and inflammasome assembly.

Beyond its effects on inflammasome assembly, DL-lactic acid was also reported to promote cytokine processing through a mechanistically distinct process under extreme cell-free conditions. At approximately pH 3.4, lactic acid directly cleaved pro-IL-1β and pro-IL-18 into mature-like fragments independently of NLRP3, ASC, and caspase-1. Mass spectrometry identified Asp116, the canonical caspase-1 cleavage site, as the site of lactic acid-mediated pro-IL-1β cleavage [77]. Because this phenomenon was observed under severe acidification in a cell-free system, it should be distinguished from caspase-1-dependent cytokine maturation in intact cells and should not itself be interpreted as NLRP3 inflammasome activation.

Collectively, these studies indicate that endogenous lactic acid fermentation and intracellular lactic acidification can promote NLRP3 inflammasome activation in defined metabolic and acidic contexts. Interpretation of these findings requires explicit distinction between endogenous production and exogenous administration, lactic acid and lactate salts, extracellular and intracellular pH, and intact-cell inflammasome signaling and cell-free chemical cleavage. Accordingly, the pro-inflammatory effects described in this section should not be generalized to all forms of lactate administration or all physiological contexts.

## 5. Lactate-Mediated Inflammatory Restraint, Tissue Repair, and Tumor-Associated Immunosuppression

**Figure 2:**
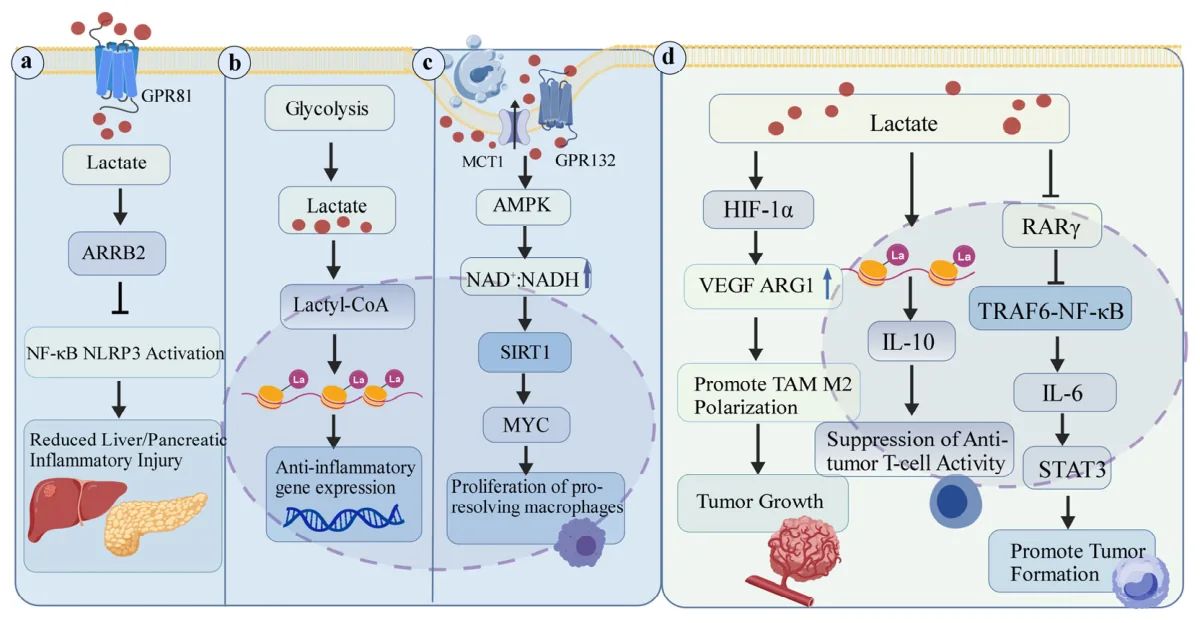
Lactate pathways in inflammatory restraint, repair and tumor-associated immunosuppression. (**a**) GPR81/ARRB2-dependent signaling restrains macrophage inflammatory priming under pH-controlled conditions. (**b**) Histone lactylation induces anti-inflammatory gene expression. (**c**) Intracellular lactate supports continual efferocytosis; exported lactate supports pro-resolving macrophage expansion. (**d**) Tumor pathways distinguish macrophage remodeling from macrophage-to-cancer-cell signaling. See Section 5.1, Section 5.2, Section 5.3 and Section 5.4 for experimental contexts and evidence limitations. Created with BioGDP (https://biogdp.com/), accessed on 9 October 2026.

The effects considered in this section do not represent a single anti-inflammatory program. Under pH-controlled conditions, extracellular lactate can restrain innate inflammatory priming through GPR81/HCAR1. At later stages of inflammatory activation or during efferocytosis, lactate can support homeostatic transcription, continual apoptotic-cell clearance, and the expansion of pro-resolving macrophages. In tumors, however, related homeostatic and repair-associated programs can be co-opted to suppress antitumor immunity. The biological outcome therefore depends on the inflammatory phase, lactate source and compartment, recipient cell, and downstream receptor or chromatin pathway (Figure 2).

### 5.1. GPR81/HCAR1-Mediated Suppression of Innate Immune Priming

Because extracellular acidification can potentiate NLRP3 inflammasome responses, the inhibitory effects of lactate reported by Hoque et al. [20] should be distinguished from the potentiating effects of acidifying lactic acid treatments described in Section 4.4. In their study, sodium L-lactate was administered in medium buffered at pH 7.4, thereby minimizing extracellular acidification as a potential confounding factor. Under these conditions, sodium L-lactate inhibited LPS-induced nuclear factor κB (NF-κB) activation in mouse macrophages and reduced the LPS-induced mRNA expression of *Il1b*, *Nlrp3*, and *Casp1*. Similar transcript-level effects were observed in adherent human peripheral blood mononuclear cells [20]. Mechanistically, GPR81/HCAR1, a cell-surface G-protein-coupled receptor for lactate, can signal through β-arrestin 2 (ARRB2)-dependent pathways [20,73,78]. In macrophages, silencing either *Gpr81* or *Arrb2* abolished the lactate-mediated suppression of LPS-induced *Il1b* expression, supporting a functional requirement for both proteins in this inhibitory response. Sodium L-lactate also reduced caspase-1 cleavage and IL-1β release, indicating attenuation of downstream inflammasome output [20]. Together, these findings support a GPR81-ARRB2-dependent inhibitory mechanism through which extracellular lactate restrains TLR-dependent inflammatory priming and downstream inflammasome responses in this experimental setting (Figure 2a).

### 5.2. Delayed Histone Lactylation Supports Reparative Transcription

In the original lactate-clock model, histone lactylation represents a delayed metabolic–epigenetic mechanism through which glycolysis-derived lactate accumulation can shape macrophage transcription after the initial pro-inflammatory response [11,40,79]. In macrophages challenged with LPS/interferon-γ (IFN-γ) or bacteria, histone lactylation increased predominantly at later time points, after the induction of early inflammatory genes. Increased H3K18la was associated with late expression of homeostatic genes, including *Arg1*. Deletion of *Ldha* or inhibition of glycolysis reduced lactate production, histone lactylation, and *Arg1* expression, whereas exogenous lactic acid increased these readouts [14]. In vitro reconstitution assays further showed that p300 can install lactylation marks on histones using lactyl-CoA as the donor, demonstrating writer activity at the biochemical level without establishing locus-specific causality in intact macrophages [14]. These findings gave rise to the concept of an endogenous “lactate clock” (Figure 2b) [14,80]. However, the causal relationship between histone lactylation and repair-associated gene expression remains contested. In LPS-stimulated BMDMs, Dichtl et al. [81] found that *Arg1* induction required IL6/IL6R signaling, while JAK2 inhibition and STAT3 phosphorylation supported downstream JAK-STAT3 involvement. *Arg1* could be dissociated from bulk Kla signals, broader M2-associated transcription was not consistently induced, and IL6 plus lactate was insufficient without additional inflammatory inputs [81]. Thus, the concurrent increases in lactate, histone lactylation and *Arg1* expression do not by themselves establish a locus-specific causal role for H3K18la in this experimental setting [40,82,83].

Complementary evidence links this delayed metabolic–epigenetic response to tissue recovery. In macrophages, deficient in B-cell adaptor for phosphoinositide 3-kinase (BCAP), impaired glycolysis and lactate production were accompanied by reduced histone lactylation, defective induction of reparative genes, prolonged intestinal inflammation, and impaired recovery from experimental colitis [60]. After a short TLR pulse and washout, sodium lactate restored histone lactylation and selected reparative transcripts in BCAP-deficient BMDMs, providing metabolic rescue evidence while leaving histone-mark-specific mediation unresolved [60]. BCAP also regulates FOXO1 and GSK3β, indicating that histone lactylation is one component of a broader reparative program rather than the sole downstream mechanism. Genome-wide analyses further showed that H3K18la is enriched at active promoters and tissue-specific enhancers [82]. During ischemia-induced muscle regeneration, changes in macrophage H3K18la between days 2 and 4 predicted later transcriptional changes, although this temporal association did not establish lactylation dependence [84]. Stronger perturbational evidence was obtained in atherosclerosis, where macrophage MCT4 deficiency or inhibition reduced lactate efflux, increased intracellular H3K18la, activated anti-inflammatory and repair-associated genes, and ameliorated disease [85]. Collectively, these studies support the contribution of histone lactylation to reparative transcription, while also showing that the strength of causal evidence varies among models (Table 1).

### 5.3. Efferocytosis-Induced Lactate Supports Continual Clearance and Pro-Resolving Macrophage Expansion

Efferocytosis, the phagocytic clearance of apoptotic cells, elicits a glycolytic response that differs in its trigger, kinetics, and downstream function from the sustained glycolytic program of inflammatory macrophages [56]. Morioka et al. [21] showed that apoptotic-cell uptake activates an SLC-dependent program involving increased glucose uptake and SLC16A1-mediated lactate export. Glycolysis supported actin polymerization and continued apoptotic-cell uptake, whereas exported lactate promoted anti-inflammatory gene expression in surrounding macrophages. Schilperoort et al. [56] further showed that uptake of an initial apoptotic cell rapidly activates PFKFB2, producing a transient glycolytic increase distinct from pro-inflammatory macrophage glycolysis. The resulting intracellular lactate increased the calcium-dependent availability of the efferocytosis receptors MerTK and LRP1 at the cell surface without altering their total abundance. This response facilitated subsequent apoptotic-cell uptake and thereby sustained continual efferocytosis. Thus, intracellular lactate supported repeated clearance by regulating receptor availability rather than by acting as an extracellular receptor ligand.

Lactate also contributes to efferocytosis-induced macrophage proliferation, a related but mechanistically distinct process that expands the pool of pro-resolving macrophages. Gerlach et al. [86] showed that nucleotides generated through DNase2a-dependent degradation of apoptotic-cell DNA activate DNA-PKcs-mTORC2/Rictor signaling and increase *Myc* transcription. Ngai et al. [18] subsequently identified a complementary metabolic input. Lactate exported through SLC16A1 signaled through GPR132-PKA-AMPK, increased the NAD^+^/NADH ratio, activated SIRT1, and stabilized Myc protein [18]. Integration of these transcriptional and post-transcriptional inputs enabled macrophage proliferation and supported tissue repair. Taken together, efferocytosis-induced glycolysis generates lactate with compartment-specific functions. Intracellular lactate sustains continual apoptotic-cell uptake, whereas exported lactate promotes anti-inflammatory transcription in surrounding macrophages and the GPR132-dependent expansion of pro-resolving macrophages (Figure 2c).

### 5.4. Lactate-Driven Macrophage Remodeling and Immune Suppression in Tumors

In chronic high-lactate tumor microenvironments, pathways associated with homeostasis and tissue repair can be redirected toward immune suppression [87]. Colegio et al. [15] showed that tumor-derived lactic acid induced *Vegf* and *Arg1* expression in tumor-associated macrophages through HIF-1α-dependent mechanisms, thereby promoting angiogenesis and tumor growth. Lactate-driven macrophage immunosuppression can also arise from macrophage-intrinsic metabolism. In glioblastoma, tumor-derived soluble factors activated the PERK-ATF4-GLUT1 pathway in monocyte-derived macrophages, increasing glycolysis and intracellular lactate production. Lactate-dependent histone lactylation promoted IL-10 expression and suppression of T-cell activity, whereas myeloid-lineage PERK deletion reduced histone lactylation, increased intratumoral T-cell accumulation, and delayed tumor growth [16]. Beyond macrophage-intrinsic lactate production, tumor-derived lactate can directly reprogramme macrophage chromatin. In colorectal cancer, Li et al. [88] found that tumor-derived lactate promoted H3K18 lactylation and repressed *RARG* transcription in macrophages. Reduced RARγ expression released its inhibitory effect on TRAF6, enhancing NF-κB-dependent IL6 production in macrophages and subsequent STAT3 activation in colorectal cancer cells (Figure 2d). Lactate can also suppress macrophage-mediated tumor clearance by reprogramming tumor-cell chromatin. In glioblastoma, lactate produced by glioblastoma stem cells and microglia/macrophages induced histone lactylation in tumor cells and increased CD47 expression through a CBX3-EP300-associated mechanism, thereby limiting macrophage phagocytosis [89]. Collectively, these studies indicate that lactate can contribute to tumor immune evasion both by reprogramming macrophages toward tumor-supportive states and by reducing tumor-cell susceptibility to macrophage-mediated clearance.

## 6. Controversies, Methodological Challenges, and Translational Prospects

Lactate influences macrophages through changes in carbon use and redox balance, extracellular receptor signaling, and covalent lactyl-group formation [14,18,20,24,25]. These roles are related but not interchangeable. Their consequences depend on source, chemical form, concentration, pH, compartment, timing, and cell state. Table 2 summarizes the principal experimental contexts, proposed mechanisms, biological outcomes, and evidence limitations discussed in this review.

### 6.1. Experimental Definition and Mechanistic Attribution of Lactate Exposure

A major source of inconsistency is that chemically and physiologically distinct interventions are often grouped under the term “lactate treatment”. Exogenously added lactic acid and sodium L-lactate differ in proton load and final extracellular pH, and even the effects of sodium lactate can change with the pH of the medium. Acidifying lactic-acid preparations have been associated with inflammasome activation or potentiation, whereas sodium L-lactate under pH-controlled conditions can suppress innate immune priming through GPR81/HCAR1 [19,20,77]. Endogenous glycolytic lactate accumulation is also not equivalent to extracellular exposure because it is coupled to broader changes in glycolytic flux, NAD^+^/NADH balance, and pyruvate metabolism [17,24,25]. Future studies should therefore report the chemical formulation, concentration, exposure duration, initial and final extracellular pH, and whether intracellular acidification occurs. They should also distinguish endogenous production from extracellular addition and quantify intracellular and extracellular lactate whenever the proposed mechanism depends on transport or compartmentalization.

Even when these experimental variables are defined, the responsible mechanism may remain difficult to assign. Extracellular lactate can activate cell-surface receptors, enter cells through proton-coupled monocarboxylate transporters, and influence metabolism or intracellular pH. Dependence on a receptor or adaptor does not exclude simultaneous contributions from lactate transport and metabolism, nor does it establish direct physical binding between signaling proteins. For example, Hoque et al. [20] showed that GPR81 and ARRB2 were functionally required for the suppression of LPS-induced pro-IL-1β expression and co-immunoprecipitated within the same complex. However, a recent enhanced bystander bioluminescence resonance energy transfer (ebBRET) study detected GPR81-mediated G-protein activation without ligand-induced β-arrestin recruitment [90]. These findings may differ because the studies used distinct cellular systems, ligands, and assays. They support an ARRB2-dependent inhibitory mechanism in the macrophage model studied by Hoque et al. [20], but do not establish ligand-induced GPR81-ARRB2 recruitment as a general property of the receptor. More broadly, future macrophage studies should separately test GPR81 dependence, MCT-mediated uptake, and pH effects to determine whether a given lactate response is mediated by receptor signaling, intracellular metabolism, or acidification.

### 6.2. Lactylation Chemistry and Enzymology Remain Context-Dependent

Lysine L-lactylation and D-lactoylation are stereochemically distinct modifications that can arise through different donor chemistries and should not be treated as interchangeable. Glycolysis-associated histone Kla has been linked to context-dependent macrophage responses, ranging from delayed homeostatic transcription after acute activation to enhanced pro-inflammatory responsiveness in trained immunity [14,83]. In another model, GLO2 loss increased LGSH, lactoyl-CoA, selected histone Kla marks, and inflammatory responses. Although LGSH can provide a route to D-lactoylation, the histone marks were not stereochemically resolved, and the pro-inflammatory phenotype was not specifically attributed to D-lactoylation [35]. By contrast, directly identified LGSH-induced D-lactoylation of RelA at K310 attenuated NF-κB signaling [23]. These findings indicate that neither stereochemistry nor donor origin predicts a uniform macrophage response; functional attribution additionally requires identification of the modified substrate and lysine site.

This functional heterogeneity is compounded by uncertainty over how individual lactylation events are installed and removed. Nuclear GTPSCS and ACSS2 have been reported to generate lactyl-CoA in specific tumor models, coupled respectively to p300- and KAT2A-dependent histone lactylation, whereas LGSH provides a non-enzymatic route to lysine lactoylation [32,33,34,35]. HDAC1-3 and SIRT2 have established delactylase activity, yet recent biochemical and cellular evidence indicates that class I HDACs can also catalyze lactate-dependent lysine lactylation under particular conditions [37,38,39]. Thus, the conventional separation of lactylation enzymes into fixed “writers” and “erasers” is likely to be conditional rather than absolute. The relative contribution of these routes in macrophages remains uncertain and may vary by subcellular compartment, substrate, and inflammatory state. Stable-isotope tracing, stereospecific mass spectrometry, compartment-resolved metabolomics and enzyme-specific perturbation with rescue are needed to define which route generates each functional lactylation event.

Analytical specificity is essential before assigning a lactylation mechanism. A pan-Kla immunoblot does not by itself establish all modified proteins and lysine sites; stereochemical assignment additionally requires validated isomer selectivity. Histone-site antibodies should be validated using cognate and competing modified peptides, with appropriate loading and chromatin controls [14,22]. L-lactyllysine, D-lactyllysine, and Nε-(carboxyethyl)lysine are isomeric lysine modifications sharing a +72.021-Da mass increment; high mass accuracy alone cannot distinguish them. Isomer-specific antibodies and chiral derivatization coupled to chromatography and MS provide complementary ways to address this ambiguity [22]. Site assignments should include informative fragmentation spectra and comparison with synthetic standards. Targeted analyses using isotope-labeled L- and D-lactylated peptides demonstrated sequence-dependent chromatographic separation and both stereoisomers on mouse testis histones [91]. Relative changes in enriched peptide intensity should be distinguished from modification stoichiometry, with normalization to parent protein or histone abundance and, where feasible, internal standards. Chemical confirmation identifies a modified substrate; assigning its regulated genes or function additionally requires appropriate locus- or substrate-resolved measurements and perturbations that address alternative explanations.

### 6.3. Chromatin Association Does Not Establish Locus-Specific Causality

A separate challenge is distinguishing chromatin association and temporal prediction from causal transcriptional regulation by histone lactylation. The original lactate-clock model linked late lactate accumulation, histone lactylation, and the induction of homeostatic genes such as *Arg1* [14]. However, parallel changes in lactate, histone lactylation, and gene expression do not establish that a defined lactylation mark at a specific genomic locus is necessary or sufficient for the associated transcriptional response. Dichtl et al. [81] found that LPS-induced *Arg1* expression depended on autocrine/paracrine IL6-IL6R-STAT3 signaling and could be dissociated from bulk Kla signals in their macrophage model. Genome-wide studies further found that H3K18la is enriched at active promoters and enhancers and that changes in its genomic distribution can predict subsequent transcriptional changes [82,84]. These observations establish chromatin association and temporal precedence, but not locus-specific necessity or sufficiency. Perturbational studies in atherosclerosis, trained immunity, and tumor models provide stronger evidence that histone lactylation can contribute functionally in specific contexts [83,85,88,89]. However, because many of these interventions alter lactate production, transport, or enzymatic activity more broadly, they do not uniformly isolate the causal contribution of a defined histone residue at a single genomic locus. The central unresolved question is therefore whether a defined histone lactylation mark at a specific genomic locus is necessary, sufficient, or both for a given transcriptional response, and how this relationship varies across macrophage states.

Future causal studies should combine orthogonal perturbations that control for changes in pH and global metabolism with residue-specific and locus-resolved chromatin analyses. Manipulating glycolysis, LDHA, MCTs, or p300 can alter multiple metabolic and signaling pathways beyond lactylation. Residue substitution also requires controls: a lysine-to-arginine mutation prevents other modifications of that lysine and is not lactylation-specific. Occupancy maps generated by CUT&Tag or ChIP should therefore be paired with perturbational tests of necessity and, where feasible, sufficiency or rescue. The development of tools that selectively remove a defined lactylation mark from a specific locus would enable tests of necessity, whereas locus-targeted installation of the same mark without broad metabolic manipulation would provide the most direct test of sufficiency. Single-cell and spatial approaches can then localize the associated chromatin and transcriptional programs to relevant macrophage subsets and tissue niches, although causal attribution would still require cell-type-restricted perturbation.

### 6.4. Translational Strategies Require Stage- and Cell-Specific Targeting

These mechanistic distinctions have important implications for therapeutic design. Broadly increasing or decreasing lactate is unlikely to achieve therapeutic selectivity because lactate-associated pathways can exacerbate inflammatory injury in some settings, while supporting resolution and tissue repair or sustaining tumor-associated immune suppression in others. In preclinical models of sepsis and acute inflammatory injury, intracellular lactate-ROS signaling and lactate-associated HMGB1 modification, release, and downstream inflammatory activity have emerged as candidate intervention points [17,30,73,75,92]. During resolution, suppression of lactate production could disrupt continual efferocytosis and the expansion of pro-resolving macrophages [18,56]. Transporter-directed interventions require additional caution because their effects depend on transporter identity and flux direction: SLC16A1-mediated lactate export contributes to an anti-inflammatory tissue environment, whereas MCT4 inhibition can increase intracellular H3K18la and reparative gene expression [21,85]. In tumors, preclinical studies suggest that targeting sustained lactate production or downstream lactylation-associated immune programs may limit immune evasion, although the relevant cell type and mechanism differ across tumor contexts [16,88,89].

Any candidate lactate-directed strategy should therefore define the disease stage, lactate source, responding cell, relevant compartment, and intended downstream effector. Future preclinical studies should pair pharmacological or genetic perturbation with, where relevant, measurements of lactate flux, extracellular and intracellular pH, receptor and transporter dependence, lactylation chemistry and cell-type-specific functional outcomes. Such designs can help identify pathogenic processes whose inhibition improves outcome while revealing lactate-dependent functions that may need to be preserved during host defense, resolution and tissue repair. This context-selective approach provides a more defensible basis for translation than treating lactate or global Kla as uniformly harmful or beneficial targets.

Therapeutic approaches act at different levels: lactate production, transport, receptor signaling, or modification chemistry. Inhibiting glycolysis or LDH changes carbon flux and redox balance; transporter inhibition changes lactate distribution and may either reduce uptake or retain intracellular lactate; receptor modulation targets extracellular sensing; and donor-producing or histone-modifying enzymes have additional metabolic and chromatin functions [17,18,32,33,37,38,39,85]. These differences preclude interpreting all interventions as equivalent lactylation therapies. In advanced cancer, a phase I dose-escalation study of the oral MCT1 inhibitor AZD3965 established a recommended phase II dose, provided evidence of target engagement, and reported dose-limiting asymptomatic ocular changes, troponin elevation, and acidosis. It did not establish macrophage-directed clinical efficacy [93]. AZD3965 does not inhibit MCT4, making transporter expression and compensatory transport relevant to patient selection and response. Receptor targeting also requires safety evaluation: GPR81 agonism increased blood pressure and reduced renal blood flow and glomerular filtration in male mice, with receptor-dependent effects, providing a preclinical safety signal rather than demonstrated human toxicity [94]. Local or macrophage-directed delivery may improve selectivity, but this remains a strategy requiring evaluation. Further studies should compare therapeutic benefit with effects on antimicrobial defense, efferocytosis, tissue repair, and systemic metabolism.

## 7. Conclusions

The central conclusion of this review is that lactate does not impose a fixed pro-inflammatory or anti-inflammatory state on macrophages. Its effects depend on the temporal phase, cellular source and compartment, chemical formulation and pH, receptor and transporter engagement, metabolic fate, lactyl-group donor chemistry, modified protein and lysine site, and disease microenvironment. This mechanism-resolved framework helps explain how lactate-associated pathways can amplify acute inflammatory injury, support resolution and tissue repair, or sustain tumor-associated immune suppression. Future studies should quantify lactate production, uptake, efflux, and reutilization while experimentally separating receptor signaling, MCT-mediated transport, intracellular acidification, metabolic conversion, inflammasome regulation, and the stereochemical and site specificity of lactylation. These mechanisms should be tested in defined macrophage subsets, tissue niches and disease stages using orthogonal perturbations and cell-type-specific functional readouts. Such an approach may identify intervention points that limit pathological inflammation or tumor immune evasion while preserving host defense, resolution and tissue repair.

## Figures and Tables

**Table 1:** Evidence supporting and qualifying lactate-clock interpretations.

Study/Setting	Main Evidence	Limit or Competing Explanation
Zhang et al. [14] (2019); stimulated BMDMs	*Ldha* loss, metabolic inhibition and lactic-acid supplementation changed Kla/*Arg1*; reconstituted chromatin supported transcription.	These interventions do not isolate *Arg1*-locus H3K18la.
Irizarry-Caro et al. [60] (2020); BMDMs/colitis	BCAP loss impaired Kla and reparative readouts; sodium lactate rescued selected transcripts.	BCAP signaling and other lactate effects remain alternatives.
Dichtl et al. [81] (2021); LPS-stimulated BMDMs	*Il6/Il6ra* loss and JAK2/pSTAT3 manipulation supported cytokine-dependent *Arg1*; *Arg1* dissociated from bulk Kla.	IL6 plus lactate is insufficient to induce *Arg1*; additional inflammatory inputs required.
Desgeorges et al. [84] (2024); ischemic-muscle macrophages	Day 2–4 H3K18la changes predicted day 4–7 expression.	Temporal prediction does not establish lactylation dependence.
Zhang et al. [85] (2024); MCT4/atherosclerosis	MCT4 perturbation linked H3K18la, reparative transcription and disease improvement.	Transport/metabolic perturbation alone does not isolate histone-mark mediation.

BMDMs, bone marrow-derived macrophages; BCAP, B-cell adaptor for phosphoinositide 3-kinase; Kla, lysine lactylation. Histone residue specificity and genomic locus specificity are distinct questions.

**Table 2:** Context-dependent effects of lactate metabolism and lactylation on macrophage responses.

Context	Experimental Condition	Mechanism	Biological Outcome	Key Limitations	Ref.
Temporal phase					
Early infection/sepsis	SPNS2-deficient macrophages; excessive PGE2-EP4 signaling during early infection	PGE2-EP4 ↑ → malate-aspartate shuttle activity ↓ and mitochondrial respiration ↓ → intracellular lactate ↑ and mtROS ↑	Pro-inflammatory: early hyperinflammation and organ injury	Specific to SPNS2/S1P disruption; lactylation was not tested as a required mediator	[17,30]
Later phase of activation/tissue repair	Delayed H3K18la accumulation after inflammatory activation	H3K18la enrichment at active promoters and enhancers	Concurrent repair-associated gene expression (e.g., *Arg1*, *Vegfa*)	Timing or occupancy alone does not prove locus-specific causality; *Arg1* may be IL6-STAT3-dependent ^a^	[14,82,85]
Cellular source, recipient, and compartment					
Tumor → macrophage	Tumor-derived extracellular lactic acid	HIF-1α → VEGF production and an ARG1-positive TAM program	Tumor-promoting: angiogenesis and increased tumor growth	Low pH alone did not induce *Vegfa*, but lactic acid was not compared with pH-matched sodium L-lactate	[15]
Macrophage-intrinsic (tumor)	Tumor factors → macrophage PERK-ATF4-GLUT1; macrophage-intrinsic glycolysis	Intracellular lactate ↑ → histone Kla → IL-10 expression	Tumor-immunosuppressive: T-cell suppression and tumor growth	Macrophage-intrinsic program; distinct from tumor-cell relay	[16]
Macrophage → endothelium/neutrophil	Macrophage-derived, exosome-associated HMGB1	HMGB1 lactylation/acetylation → exosomal relay	Pro-inflammatory: endothelial barrier disruption and neutrophil NET formation	Endothelial and neutrophil outcomes derive from separate sepsis models; HMGB1 acetylation co-occurs in the endothelial study	[73,75]
Macrophage/tumor → glioblastoma tumor cell	Tumor- or myeloid-derived lactate acting on glioblastoma cells	CBX3/EP300-supported histone Kla → tumor-cell CD47 ↑	Tumor-immunosuppressive: decreased macrophage/microglial phagocytosis	CD47 is induced in tumor cells, not macrophages	[89]
Chemical form, donor chemistry, and pH					
Acidifying lactic acid	Exogenous lactic acid; final pH 6.6 to 6.9	Intracellular acidification → mtROS and PKR-dependent NLRP3 assembly	Pro-inflammatory: enhanced caspase-1 activation and IL-1β release	Effect inseparable from proton load; does not isolate lactate-anion signaling	[19,77]
Sodium lactate (pH control)	Exogenous sodium lactate at pH 7.4; nigericin-stimulated macrophages	No comparable cytosolic acidification or acidification-driven NLRP3 potentiation	No acidification-associated increase in IL-1β release	Tests proton load, not HCAR1-dependent restraint of TLR priming	[77]
Endogenous fermentation	PKM2-dependent glycolysis coupled to LDH-mediated fermentation	Fermentative flux → PKR phosphorylation → NLRP3 and AIM2 inflammasome support	Pro-inflammatory: IL-1β and IL-18 processing	PKM2 or LDH perturbation alters broader glycolytic flux, pyruvate handling, and redox balance and therefore does not isolate lactate as the causal mediator	[19,76]
LGSH/GLO2 loss	GLO2-knockout RAW264.7 macrophages; Kdo2-lipid A stimulation	GLO2 loss → LGSH/lactoyl-CoA accumulation → increased histone Kla, accompanied by altered chromatin accessibility	Pro-inflammatory: amplified TLR4-agonist response	Histone stereochemistry and causal site-specific necessity were unresolved	[35]
SLG/RelA K310	S-D-lactoylglutathione; site-resolved D-lactoylation	RelA K310 D-lactoylation → reduced NF-κB activity	Anti-inflammatory: feedback restraint of innate activation	Target-specific; not a global D-lactoylation effect	[23]
Receptors and transporter direction					
HCAR1/GPR81	pH-neutral sodium L-lactate in LPS-treated macrophages/monocytes	HCAR1- and ARRB2-dependent suppression of inflammatory priming	Anti-inflammatory: reduced NF-κB activation and pro-IL-1β expression	Functional dependence does not prove ligand-induced ARRB2 recruitment ^b^	[20,90]
MCT-dependent uptake	Extracellular lactate → macrophage; sepsis HMGB1 model	MCT-dependent uptake supports HMGB1 lactylation; pharmacologically implicated GPR81 signaling contributes to acetylation during exosomal release	Pro-inflammatory: increased circulating exosomal HMGB1 and endothelial permeability	Inhibitor data support uptake requirement but do not establish a universal MCT isoform	[73]
SLC16A1-mediated export	Efferocytosing macrophage → extracellular lactate	Net export → GPR132 → PKA → AMPK → NAD^+^:NADH ratio ↑ → SIRT1 → Myc stabilization	Pro-resolving: macrophage proliferation and tissue repair	Net export is context-specific; the lactate arm stabilizes Myc, whereas apoptotic-cell nucleotides induce *Myc* transcription ^c^	[18,21]
MCT4-mediated efflux	Glycolytic macrophage; MCT4 inhibition in atherosclerosis	Reduced efflux → intracellular lactate retention → p300-mediated H3K18la → anti-inflammatory and TCA-cycle gene expression	Reparative/atheroprotective: reduced atherosclerotic lesion burden	Macrophage- and atherosclerosis-specific; MCT4 blockade alone does not isolate the contribution of H3K18la at a defined residue or genomic locus.	[85]

Note: Rows show the dominant outcome in each cited model, not an intrinsic effect of lactate. LGSH and SLG both denote S-D-lactoylglutathione; divergent outcomes reflect differences in target proteins, modified sites, and experimental models. ^a^ In one LPS model, *Arg1* induction depended on autocrine/paracrine IL6-IL6R-STAT3 signaling and was not universally coupled to histone lactylation [81]. ^b^ HCAR1 ligands activated G proteins without detectable β-arrestin recruitment in an ebBRET assay [90]; ARRB2 dependence in macrophages may therefore be context- or assay-specific [20]. ^c^ Apoptotic-cell nucleotides increased *Myc* transcription via DNA-PKcs-mTORC2/Rictor [86], whereas lactate export-GPR132 signaling stabilized Myc protein [18]; these are complementary inputs.

## Data Availability

No new data were created or analyzed in this study. Data sharing is not applicable to this article.

## Abbreviations

2-DG: 2-deoxy-D-glucose; ACSS2: acyl-coenzyme A synthetase short-chain family member 2; AIM2: absent in melanoma 2; AMPK: AMP-activated protein kinase; ARG1: arginase 1; ARRB2: β-arrestin 2; ASC: apoptosis-associated speck-like protein containing a caspase recruitment domain; ATF4: activating transcription factor 4; BCAP: B-cell adaptor for phosphoinositide 3-kinase; BMDMs: bone marrow-derived macrophages; CBX3: chromobox protein homolog 3; CD47: cluster of differentiation 47; cGAS: cyclic GMP–AMP synthase; ChIP: chromatin immunoprecipitation; CLP: cecal ligation and puncture; CoA: coenzyme A; COX-2: cyclooxygenase-2; CUT&Tag: cleavage under targets and tagmentation; DNA-PKcs: DNA-dependent protein kinase catalytic subunit; DNase2a: deoxyribonuclease II alpha; ebBRET: enhanced bystander bioluminescence resonance energy transfer; EP4: prostaglandin E receptor 4; FAO: fatty acid oxidation; FOXO1: forkhead box O1; GLO1: glyoxalase I; GLO2: glyoxalase II; GLUT1: glucose transporter 1; GPR81/HCAR1: G protein-coupled receptor 81/hydroxycarboxylic acid receptor 1; GPR132: G protein-coupled receptor 132; GSH: glutathione; GSK3β: glycogen synthase kinase 3 beta; GTPSCS: GTP-specific succinyl-CoA synthetase; H3K18la: histone H3 lysine 18 lactylation; H3K56la: histone H3 lysine 56 lactylation; H4K5la: histone H4 lysine 5 lactylation; HDACs: histone deacetylases; HIF-1α: hypoxia-inducible factor 1 alpha; HMGB1: high mobility group box 1; ICAM-1: intercellular adhesion molecule 1; IFN-γ: interferon gamma; IL: interleukin; iNOS: inducible nitric oxide synthase; KATs: lysine acetyltransferases; KAT2A: lysine acetyltransferase 2A; Kla: lysine lactylation; LDH: lactate dehydrogenase; LDHA: lactate dehydrogenase A; LGSH: S-D-lactoylglutathione; LRP1: low-density lipoprotein receptor-related protein 1; LPS: lipopolysaccharide; MAPK: mitogen-activated protein kinase; MCTs: monocarboxylate transporters; MerTK: MER proto-oncogene tyrosine kinase; MGO: methylglyoxal; mTORC2: mechanistic target of rapamycin complex 2; mtROS: mitochondrial reactive oxygen species; NAD^+^/NADH: oxidized/reduced nicotinamide adenine dinucleotide; NETs: neutrophil extracellular traps; NF-κB: nuclear factor kappa B; NO: nitric oxide; NOX2: NADPH oxidase 2; NLRP3: NOD-like receptor family pyrin domain-containing 3; p300/CBP: E1A-binding protein p300/CREB-binding protein; PDH: pyruvate dehydrogenase; PERK: protein kinase RNA-like endoplasmic reticulum kinase; PFKFB2: 6-phosphofructo-2-kinase/fructose-2,6-bisphosphatase 2; PGE2: prostaglandin E2; PKA: protein kinase A; PKM2: pyruvate kinase M2; PKR: protein kinase R, also termed eukaryotic translation initiation factor 2 alpha kinase 2 (EIF2AK2); RARγ: retinoic acid receptor gamma; ROS: reactive oxygen species; RUBCN: Rubicon; S1P: sphingosine-1-phosphate; SIRT1: sirtuin 1; SIRT2: sirtuin 2; SLC: solute carrier; SLC16A1: solute carrier family 16 member 1; SPNS2: Spinster homolog 2; STAT3: signal transducer and activator of transcription 3; STING: stimulator of interferon genes; TAMs: tumor-associated macrophages; TCA: tricarboxylic acid; TLRs: Toll-like receptors; TNF-α: tumor necrosis factor alpha; TRAF6: TNF receptor-associated factor 6; VE-cadherin: vascular endothelial cadherin; VEGF: vascular endothelial growth factor; YAP: Yes-associated protein.
